# Traditionally Used Plants in the Treatment of Diabetes Mellitus: Screening for Uptake Inhibition of Glucose and Fructose in the Caco2-Cell Model

**DOI:** 10.3389/fphar.2021.692566

**Published:** 2021-08-20

**Authors:** Katharina Schreck, Matthias F. Melzig

**Affiliations:** Pharmaceutical Biology, Institute of Pharmacy, Freie Universitaet Berlin, Berlin, Germany

**Keywords:** plant extracts, traditional use, glucose, fructose, uptake inhibition, Diabetes mellitus, Caco2 cells

## Abstract

The traditional use of plants and their preparations in the treatment of diseases as a first medication in the past centuries indicates the presence of active components for specific targets in the natural material. Many of the tested plants in this study have been traditionally used in the treatment of Diabetes mellitus type 2 and associated symptoms in different cultural areas. Additionally, hypoglycemic effects, such as a decrease in blood glucose concentration, have been demonstrated *in vivo* for these plants. In order to determine the mode of action, the plants were prepared as methanolic and aqueous extracts and tested for their effects on intestinal glucose and fructose absorption in Caco2 cells. The results of this screening showed significant and reproducible inhibition of glucose uptake between 40 and 80% by methanolic extracts made from the fruits of *Aronia melanocarpa*, *Cornus officinalis*, *Crataegus pinnatifida*, *Lycium chinense*, and *Vaccinium myrtillus*; the leaves of *Brassica oleracea*, *Juglans regia*, and *Peumus boldus*; and the roots of *Adenophora triphylla*. Furthermore, glucose uptake was inhibited between 50 and 70% by aqueous extracts made from the bark of *Eucommia ulmoides* and the fruit skin of *Malus domestica*. The methanolic extracts of *Juglans regia* and *Peumus boldus* inhibited the fructose transport between 30 and 40% in Caco2 cells as well. These findings can be considered as fundamental work for further research regarding the treatment of obesity-correlated diseases, such as Diabetes mellitus type 2.

## Introduction

In the last years, not only did the absolute number of people suffering from Diabetes mellitus increase from 108 Mio in 1980 to 422 Mio in 2014 but also the prevalence of this disease rose from 4.7 to 8.5% among adults during this time period worldwide (WHO, 2019). Apart from the estimated annual cost to the world of US$ 827 billion, Diabetes is associated with premature mortality and decreased quality of life (WHO, 2016). Hyperglycemia, as one of the main symptoms for diagnosis, results from decreased insulin secretion and/or reduced insulin action and can lead to long-term damage and dysfunction of different organs (American Diabetes Associa, 2014). Particularly, the most common kind of the disease, Diabetes mellitus type 2 (American Diabetes Associa, 2014), can be prevented or delayed in its progress by a lifestyle, which includes a healthy diet, regular exercise with moderate intensity, and no tobacco use (WHO, 2019).

With a focus on the diet, not only a decrease of energy intake to reduce obesity (Grundy, 1998) but also the selection of the consumed food can improve the health status. Supplements can be used to ensure the adequate intake of certain nutrients and support patient’s therapy (Ota and Ulrih, 2017). For example, the reduction of glucose, fructose, and saturated long-chain fatty acids and the increased intake of polyunsaturated, omega-3 fatty acids, such as eicosapentaenoic and docosahexaenoic acid, play a very important role in the prevention and improvement of metabolic disorders by alleviating inflammation processes (Sears and Perry, 2015). Regarding the consumption of plant-based food, secondary metabolites from plants showed hypoglycemic properties by affecting different targets, for example, modulation of intracellular insulin signaling pathways, increase in insulin secretion from β-cells, and inhibition of intestinal enzymes and transporters (Hanhineva et al., 2010; Bahadoran et al., 2013; Ota and Ulrih, 2017). In order to treat obesity as one of the main risk factors for Diabetes mellitus type 2 (Smith, 2007), the reduction of nutritional uptake into enterocytes as the last step before energy overload affects the body is an important therapeutic approach. Furthermore, the inhibition of enzymes and transporters leads to increased concentrations of nutrients in distal sections of the small intestine, which initiates the “ileal brake.” This complex mechanism is known to influence the digestive process and ingestive behavior resulting in reduced appetite and food intake (Maljaars et al., 2008). The glucose transport in the small intestine is maintained by the cotransporter SGLT1 (sodium-glucose linked transporter 1) and GLUT2 (glucose transporter 2), which belongs to the GLUT family and facilitates diffusion processes. The intestinal fructose uptake is performed by GLUT5 (glucose transporter 5) and GLUT2 as well (Schreck and Melzig, 2018). Medicinal plants traditionally used in the treatment of diseases have been applied as candidates for drug discovery in the last decades (Fabricant and Farnsworth, 2001). Aspirin, atropine, ephedrine, digoxin, and morphine are examples of effective substances, which owe their discovery to investigative studies of folk medicine (Gilani and Atta-ur-RahmanTrends, 2005). Ethnopharmacology studies show the continuing acceptance of traditionally used plant preparations as a therapy option due to their long-term experience and marginal side effects (Fabricant and Farnsworth, 2001; Gilani and Atta-ur-RahmanTrends, 2005). The plants tested in this study were chosen due to their traditional use in some cultural areas and countries during the last centuries and their hypoglycemic effects, which were discovered in animal or human studies, as listed in Table 1. For example, in Jordan, preparations of *Allium sativum*, *Ceratonia siliqua*, *Cuminum cyminum*, *Juglans regia*, *Nigella sativa*, *Olea europaea*, *Sarcopoterium spinosum* have been used in the treatment of Diabetes mellitus (Al-Aboudi and Afifi, 2011), whereas in Congo, *Brassica oleracea* and *Citrus limon* have been prepared as folk medicine to treat Diabetes mellitus (Katemo et al., 2012). The plants *Adenophora triphylla*, *Cornus officinalis*, *Crataegus pinnatifida*, *Eucommia ulmoides*, *Lycium chinense*, *Pueraria lobata*, and *Rosa rugosa* belong to the pharmacopoeia of the Traditional Chinese Medicine (Stöger, 2009). Although *Adenophora triphylla* and *Crataegus pinnatifida* showed hypoglycemic effects in studies, the traditional use as antidiabetic remedy was not confirmed in English-language literature as shown in Table 1. According to Table 1, some of the plants, such as *Olea europaea* and *Brassica oleracea*, were used in different areas of the world supporting the assumption of efficacy in the treatment of the metabolic disorder. The plants used in this study have been already tested in animal experiments and hypoglycemic properties such as a decrease in blood glucose were confirmed as shown in Table 1, but the exact mechanisms still need to be studied. In order to determine the mode of action, methanolic and aqueous plant extracts were screened for their inhibitory activity on intestinal transporters using Caco2 cells, which originally stem from a colorectal adenocarcinoma cell line. After confluence, the cells need 15–21 days to differentiate and fully express intestinal characteristics, such as a brush border membrane with enzymes and transporters. The adenocarcinoma cell line is widely used as a model for intestinal transport studies (Chantret et al., 1988; Hidalgo et al., 1989; Jumarie and Malo, 1991; Sambuy et al., 2005). We have determined the expression of the SGLT1, GLUT2, and GLUT5 transporters in the Caco2 cells during 21 days (Schreck and Melzig, 2019) and adapted the experimental conditions to ensure valuable results.

**TABLE 1:** Selected plants, which are/were traditionally used in specific countries and their antidiabetic effects measured in studies and discussed active compounds (n.f. = not found in English-language literature for this indication; n.n. = not named).

Scientific plant name	Countries/areas with traditional use of plant preparations as antidiabetic remedy and adjuncts to conventional treatments in the therapy of Diabetes mellitus (reference)	Selected, antidiabetic effects of traditionally used plants measured in studies	Discussed active compounds	Reference for antidiabetic effects and discussed active compounds
*Adenophora triphylla* (thunb.) ADC.	n.f.	Decrease of blood glucose, inhibition of intestinal glucose absorption in rats and mice	n.n.	Sanae et al. (1996)
*Allium sativum* L.	Jordan Al-Aboudi and Afifi (2011), Morocco Idm’hand et al. (2020), India Rizvi and Mishra (2013), United Kingdom Swanston-Flatt et al. (1991)	Decrease of serum glucose and triglycerides, increase of serum insulin levels in diabetic rats	Allicin-type compounds	Eidi et al. (2006)
*Aronia melanocarpa* michx. elliott	n.f.	Decrease of serum glucose and lipids, reduced α-glucosidase activity	Anthocyanins	Banjari et al. (2017)
*Artemisia dracunculus* L.	United Kingdom Swanston-Flatt et al. (1991)	Decrease of elevated blood glucose level and blood insulin concentrations in diabetic mice	Flavonoids (luteolin, apigenin), coumarins (scopoletin), sesquiterpenoid lactones (costunolide), cinnamates	Ribnicky et al. (2006)
*Brassica oleracea* L.	Congo Katemo et al. (2012), Morocco Idm’hand et al. (2020), United Kingdom Swanston-Flatt et al. (1991)	Decrease of blood glucose in diabetic rats, decrease of blood lipids, and restoration of renal function in rats	n.n.	Kataya and Hamza (2008); Assad et al. (2014); Shah et al. (2016)
*Camellia sinensis*(L.) kuntze (Assam)	Morocco Idm’hand et al. (2020)	Decrease of blood glucose level in rats/mice, reduced α-glucosidase, α-amylase, and lipase activity	Catechins, flavanols, polysaccharides	Gomes et al. (1995); Han et al. (2011); Wang et al. (2017)
*Camellia sinensis*(L.) kuntze (Darjeeling)				
*Camellia sinensis*(L.) kuntze (gunpowder)				
*Camellia sinensis*(L.) kuntze (Sencha)				
*Ceratonia siliqua* L	Jordan Al-Aboudi and Afifi (2011), Morocco Idm’hand et al. (2020)	Decrease of blood glucose in rats, reduced α-glucosidase, α-amylase activity, inhibition of intestinal glucose transport	Polyphenolic compounds	Rtibi et al. (2017)
*Citrus limon* (L.) osbeck	Congo Katemo et al. (2012), United Kingdom Swanston-Flatt et al. (1991)	Decrease of blood glucose in rats	n.n.	Naim et al. (2012)
*Coffea arabica* L.	n.f.	Decrease of blood glucose in rats, reduced glucose uptake, stimulated insulin secretion	Caffeine, polyphenolic compounds: chlorogenic acid, quinolactones	Campos-Florian et al. (2013)
*Cornus officinalis*siebold and zucc.	China Li et al. (2004); Ma et al. (2014)	Decrease of blood glucose in mice, reduced α-glucosidase activity, increased glucose uptake in HepG2	Morroniside, loganin, ursolic acid	He et al. (2016)
*Crataegus pinnatifida* bunge	n.f.	Decrease of hyperglycemia in rats, modulation of insulin regulation, anti-obesity effect, anti-hyperlipidemia effect, reduced rats’ α-glucosidase activity	Flavonoids, hyperosid, chlorogenic acid, tripenic acids	Dehghani et al. (2019)
*Cuminum cyminum* L.	Jordan Al-Aboudi and Afifi (2011), Morocco Idm’hand et al. (2020)	Decrease of hyperglycemia, oxidative stress, and AGE formation in rats, reduced α-glucosidase activity	Cuminaldehyde, flavonoids	Jagtap and Patil (2010)
*Cynara cardunculus* L.	Morocco Idm’hand et al. (2020); Tahraoui et al. (2007)	Decrease in blood glucose and serum lipid levels in humans and rats	Flavonoids	Nazni et al. (2006); Heidarian and Soofiniya (2011)
*Eucommia ulmoides* oliv.	China, Japan, Korea He et al. (2014)	Decrease in blood glucose and increase in plasma insulin in rats, decreased plasma lipid levels in mice, reduced glycation	Flavonoids	Kim et al. (2004); Lee et al. (2005); Park et al. (2006)
*Hibiscus sabdariffa* L.	Morocco Idm’hand et al. (2020)	Decreased hyperglycemia, hyperinsulinemia, serum lipids, and AGE formations in rats	Polyphenolic compounds	Peng et al. (2011)
*Ilex paraguariensis* a. st.-hil	South America de Freitas Junior and de Almeida (2017)	Decrease of serum lipids and glucose in mice, modulation of food intake	Polyphenolic compounds, methylxanthines, saponins	Kang et al. (2012)
*Juglans regia* L.	Jordan Al-Aboudi and Afifi (2011), Morocco Idm’hand et al. (2020)	Decrease of fasting blood glucose, HbA1c, and fasting blood lipids in humans	Phenolic acids and flavonoids: 3- and 5-caffeoylquinic acids, quercetin-3-galactoside, quercetin-3-arabinoside	Hosseini et al. (2014)
*Lycium chinense* mill.	China Li et al. (2004)	Decrease of blood glucose and attenuation of dyslipidemia in rats	Polyphenolic compounds	Olatunji et al. (2017)
*Malus domestica* borkh.	Morocco Idm’hand et al. (2020)	Decrease of postprandial blood glucose in mice and humans, decrease of glucose absorption in mouse intestine, inhibition of human SGLT1 in *X. laevis* oocytes, inhibition of lipase *in vitro*, and decrease of plasma triglycerides in mice and humans	Polyphenolic compounds: quercetin, phlorizin, procyanidins	Sugiyama et al. (2007); Schulze et al. (2014)
*Melissa officinalis* L.	Iran, Turkey Shakeri et al. (2016)	Decrease of plasma glucose levels in rats, reduced α-glucosidase, α-amylase activity, decrease of HbA1c, serum triglyceride and fasting blood glucose levels in humans	Polyphenolic compounds (flavonoids), essential oils	Hasanein and Riahi (2015); Asadi et al. (2019)
*Mentha aquatica* L.	n.f.	Decrease of fasting blood glucose and lipid levels, nephroprotective, reduced HbA1c, and increase of insulin levels in rats	Polyphenolic compounds (flavonoids, tannins), saponins, volatile oils	Yellanur Konda et al. (2020)
*Momordica charantia* L.	Asia, South America, East Africa Rizvi and Mishra (2013), United Kingdom Swanston-Flatt et al. (1991)	Decrease of blood glucose and glycosylated haemoglobin and increase of plasma insulin in animal studies, inhibition of intestinal transporters	Charantin, polypeptide-p, momordin ic, oleanolic acid 3-O-monodesmoside, and oleanolic acid 3-O-glucuronide	Grover and Yadav (2004)
*Nigella sativa* L.	Jordan Al-Aboudi and Afifi (2011), Morocco Idm’hand et al. (2020)	Decrease of fasting blood glucose, reduced insulin resistance, and improved β-cell function	n.n.	Bamosa et al. (2010)
*Olea europaea* L.	Jordan Al-Aboudi and Afifi (2011), Morocco Idm’hand et al. (2020), Portugal Neves et al. (2009)	Decrease of serum blood glucose, triglycerides, and cholesterol, increase of serum insulin levels	Oleuropeoside	Eidi et al. (2009)
*Origanum creticum* L.	Morocco Idm’hand et al. (2020)	Decrease of blood glucose, lipids, and HbA1c in rats, reduced α-amylase activity	n.n.	Prasanna et al. (2017)
*Panax ginseng* c.a. mey.	Asia Park et al. (2019)	Decrease of 75 g-OGTT-plasma-glucose indices, fasting plasma insulin, and 75 g-OGTT-plasma-insulin indices in humans	Ginsenosides: PPT, (20R)-PPD, Rg1, Rc, Rd, Re, Rf, Rg2, Rh1, Rb1, and Rb2; peptidoglycan: panaxan B	Vuksan et al. (2008)
*Peumus boldus* molina	n.f.	Decrease of plasma glucose level, inhibition of α-amylase and lipase	Boldine	Jang et al. (2000); Buchholz and Melzig (2016)
*Potentilla aurea* L.	n.f.	Inhibition of α-amylase and lipase	n.n.	Buchholz and Melzig (2016)
*Pueraria lobata* (willd.) ohwi	China Li et al. (2004)	Decrease of fasting blood glucose, improved glucose tolerance and insulin sensitivity in mice	Isoflavones: puerarin	Prasain et al. (2012)
*Punica granatum*L. (*peel*)	Morocco Idm’hand et al. (2020); Tahraoui et al. (2007)	Decrease of fasting blood glucose and serum lipids in rats	Phenolic compounds	Bagri et al. (2009)
*Rosa rugosa* thunb.	Korea Lee et al. (2008)	Decrease of blood glucose, serum insulin, serum lipids, increased insulin sensitivity in rats, reduced α-glucosidase activity	Polyphenolic compounds	Liu et al. (2017)
*Rosmarinus officinalis* L.	Morocco Idm’hand et al. (2020); Tahraoui et al. (2007)	Decrease of blood glucose and increase of serum insulin in rabbits, reduced α-glucosidase activity	Volatile oils	Bakırel et al. (2008)
*Salvia officinalis* L.	Morocco Idm’hand et al. (2020); Tahraoui et al. (2007), United Kingdom Swanston-Flatt et al. (1991)	Decrease in 2-h-postprandial blood glucose and cholesterol in humans, decrease in serum glucose and lipids in rats	Flavonoids (rosmarinic acid, phenolic acids, carnosic compounds)	Eidi and Eidi (2009); Behradmanesh et al. (2013)
*Sarcopoterium spinosum* (L.) spach	Jordan Al-Aboudi and Afifi (2011), Israel Bachrach (2007)	Improved glucose tolerance in mice, insulin-like effects, increased insulin secretion *in vitro*, reduced α-glucosidase and α-amylase activity	Polyphenolic compounds: catechin, epicatechin	Smirin et al. (2010); Elyasiyan et al. (2017)
*Syzygium aromaticum*(L.) merr. and l.m.perry	Morocco Idm’hand et al. (2020)	Decrease of postprandial hyperglycemia in rats, reduced α-glucosidase and α-amylase activity, downregulation of intestinal transporters	Triterpenes (oleanolic acid, maslinic acid)	Khathi et al. (2013)
*Thymus vulgaris* L.	Morocco Idm’hand et al. (2020), United Kingdom Swanston-Flatt et al. (1991)	Reduced blood glucose and serum lipids in rats	Flavonoids	Ekoh et al. (2014)
*Vaccinium myrtillus* L.	Europe Helmstaedter (2007)	Decrease of blood glucose and lipids in rats	Polyphenolic compounds	Sidorova et al. (2017)
*Vitis vinifera* L.	Morocco Idm’hand et al. (2020)	Decrease of postprandial hyperglycemia in mice, reduced α-glucosidase activity	Polyphenolic compounds: flavonoids, anthocyanins	Hogan et al. (2010)

## Materials and Methods

### Plant Extract Preparations

The methanolic and aqueous extract preparations were performed according to Buchholz (2017) with modifications. The parts of the plants, which were used to prepare the extracts, are listed in Tables 2, 3. Except for the fresh fruits of *Malus domestica*, the botanicals were obtained as dried plant material from the companies. The bulbs of *Allium sativum* , fruits of *Aronia melanocarpa* and *Vaccinium myrtillus*, fruit skin of *Punica granatum*, the herbal part of *Cynara cardunculus* and *Potentilla aurea* , leaves of *Mentha aquatica* and *Olea europaea* , roots of *Panax ginseng* and *Sarcopoterium spinosum*, and seeds of *Nigella sativa* were purchased from Kräuter Schulte aktiv Kräuter Drogerie e.K. (Gernsbach, Germany). The following herbal drugs were obtained from Alfred Galke GmbH (Gittelde, Germany): flowers of *Hibiscus sabdariffa* and *Syzygium aromaticum* ; fruits of *Ceratonia siliqua*; fruit skin of *Citrus limon*; leaves of *Brassica oleracea* , *Camellia sinensis*, *Ilex paraguariensis*, *Juglans regia*, *Melissa officinalis*, *Origanum creticum*, *Rosmarinus officinalis*, and *Salvia officinalis*; and seeds of *Cuminum cyminum* and *Vitis vinifera* (pomace). The plant materials used in Traditional Chinese Medicine, such as the bark of *Eucommia ulmoides* ; flowers of *Rosa rugosa*; fruits of *Cornus officinalis* , *Crataegus pinnatifida*, and *Lycium chinense*; and the roots of *Adenophora triphylla* and *Pueraria lobata* were acquired from Zieten Apotheke (Berlin, Germany). The fruits of the cucurbitaceae *Momordica charantia* were kindly given by Dr. Serhat Sezai Çiçek (Institute of Pharmacy of Christian-Albrechts-Universitaet Kiel, Germany). The green seeds of *Coffea arabica* were purchased from Ridders Kaffeerosterei (Berlin, Germany). The leaves of *Peumus boldus* were gained from Heinrich Klenk GmbH & Co. KG (Schwebheim, Germany), whereas the leaves of *Artemisia dracunculus* and the fresh fruits of *Malus domestica* were obtained from a local supermarket. The fresh fruits of *Malus domestica* were washed with water. The fruit’s skin was cut into small pieces, dried in a drying cabinet at 40°C for 3 days, and freeze-dried for 48 h. The dried plant material was ground with a M20 mill from IKA®-Werke GmbH & Co. KG (Staufen, Germany). 10.0 g of the plant’s powder, adjusted to the drug extract ratio as listed in Tables 2, 3, was extracted in 100 ml. For the preparation of the aqueous extracts, plant material was extracted in water obtained from the ultrapure water system LaboStar from Siemens AG Wasseraufbereitung (Barsbüttel, Germany) for 15 min at 40°C and filtered with a Buechner filter. In some cases, extracts were centrifuged at 4°C first with an Allegra X-30R centrifuge from Beckman Coulter GmbH (Krefeld, Germany) to facilitate the filtration process. The extracts were freeze-dried for at least 48 h with the lyophilization machine Alpha 2-4 LSCplus from Martin Christ Gefriertrocknungsanlagen GmbH (Osterode am Harz, Germany). The powder was stored at −20°C until use. For the preparation of the methanolic extracts, plant material was extracted in methanol purchased from VWR International S.A.S. (Fontenay-sous-Bois, France) for 60 min at 40°C and filtered. In order to obtain a dry powder, they were evaporated to 5 ml with the rotary evaporator RV 10 basic from IKA®-Werke GmbH & Co. KG (Staufen, Germany) and dried in a Petri dish from VWR International GmbH (Darmstadt, Germany) under the exhaust hood overnight. Subsequently, the extracts were freeze-dried to lose residual water and stored at −20°C until use. For application in cell experiments, the aqueous extracts were solved in DPBS (Dulbecco’s phosphate-buffered saline) purchased from Lonza Cologne AG (Cologne, Germany), whereas the extracts made with methanol were first dissolved in DMSO (0.3% V/V final well concentration), which was acquired from Fisher Scientific GmbH (Schwerte, Germany), and filled with DPBS until final volume. Extract solutions were filtered with a 0.8 µm and a 0.2 µm sterile filter purchased from Carl Roth GmbH + Co. KG (Karlsruhe, Germany) and stored at −20°C until use. Three different extract concentrations (1.0, 0.1, or 0.01 mg/ml) were tested in MTT assay [3-(4,5-dimethylthiazol-2-yl)-2,5-diphenyltetrazolium bromide] as described in the Supplementary Material and only the highest concentration, which did not affect cell viability, was used in uptake studies. The GLUT2 inhibitor phloretin, which was purchased from Cayman Chemical (Michigan, United States), and the SGLT1 inhibitor phlorizin applied from Carl Roth GmbH + Co. KG (Karlsruhe, Germany) were used with a final well concentration of 100 µM as a positive control for uptake inhibition of glucose.

**TABLE 2:** Results of glucose and fructose uptake studies with methanolic extracts, the used plant part, drug extract ratio, and tested concentration, which did not show any influence on cells in MTT assay. Only the plants that showed the strongest inhibition rates on glucose uptake were tested for fructose uptake inhibition. Regarding methanolic and aqueous extracts, the extract of a plant that showed the stronger inhibition on glucose absorption was repeated and tested for its impact on fructose uptake; mean value ± SD; *significant difference in *U*-test (*n* = 2 with 12 replicates each; two-sided; *α* = 0.05); n.d. = not determined.

Scientific plant name	Used part of the plant	Drug extract ratio for methanolic extracts	Used methanolic extract concentration in mg/mL	Uptake inhibition of glucose in %	Uptake inhibition of fructose in %
*Adenophora triphylla* (thunb.) ADC.	Root	n.d.	1	64.6* ± 4.5	<25
*Allium sativum* L.	Bulb	20:1	0.01	<25	n.d.
*Aronia melanocarpa* michx. elliott	Fruit	n.d.	1	57.1* ± 7.2	<25
*Artemisia dracunculus* L.	Leaf	9:1	0.1	<25	n.d.
*Brassica oleracea*L. “*Capitata alba*”	Leaf	3:1	1	82.8* ± 4.3	<25
*Camellia sinensis*(L.) kuntze (Assam)	Leaf	11:1	0.1	<25	n.d.
*Camellia sinensis*(L.) kuntze (Darjeeling)	Leaf	5:1	0.1	<25	n.d.
*Camellia sinensis*(L.) kuntze (gunpowder)	Leaf	5:1	0.1	<25	n.d.
*Camellia sinensis*(L.) kuntze (Sencha)	Leaf	5:1	0.1	<25	n.d.
*Ceratonia siliqua* L.	Fruit	4:1	1	<25	n.d.
*Citrus limon* (L.) osbeck	Fruit skin	7:1	0.1	<25	n.d.
*Coffea arabica* L.	Green seed	7:1	1	<25	n.d.
*Cornus officinalis*siebold and zucc.	Fruit	3:1	1	73.8* ± 6.7	<25
*Crataegus pinnatifida* bunge	Fruit	n.d.	1	73.7* ± 4.2	<25
*Cynara cardunculus* L.	Herb	n.d.	0.1	<25	n.d.
*Eucommia ulmoides* oliv.	Bark	n.d.	0.1	<30	n.d.
*Hibiscus sabdariffa* L.	Flower	4:1	0.1	<25	n.d.
*Ilex paraguariensis* a. st.-hil	Leaf	7:1	0.1	<25	n.d.
*Juglans regia* L.	Leaf	12:1	1	52.4* ± 1.7	30.2* ± 11.7
*Lycium chinense* mill.	Fruit	n.d.	1	75.2* ± 0.5	<25
*Melissa officinalis* L.	Leaf	n.d.	0.1	<25	n.d.
*Mentha aquatica* L.	Leaf	16:1	0.1	<25	n.d.
*Momordica charantia* L.	Fruit	13:1	0.1	<25	n.d.
*Nigella sativa* L.	Seed	14:1	0.1	<25	n.d.
*Olea europaea* L.	Leaf	5:1	0.1	<25	n.d.
*Origanum creticum* L.	Leaf	16:1	0.1	<25	n.d.
*Panax ginseng* c.a. mey	Root	9:1	0.1	<25	n.d.
*Peumus boldus* molina	Leaf	5:1	1	47.5* ± 7.5	32.6* ± 26.1
*Potentilla aurea* L	Herb	15:1	1	<25	n.d.
*Pueraria lobata* (willd.) ohwi	Root	6:1	0.1	<25	n.d.
*Rosa rugosa* thunb.	Flower	6:1	0.1	<25	n.d.
*Rosmarinus officinalis* L.	Leaf	6:1	0.1	<25	n.d.
*Salvia officinalis* L.	Leaf	9:1	0.1	<25	n.d.
*Sarcopoterium spinosum* (L.) spach	Root	14:1	0.1	<25	n.d.
*Syzygium aromaticum*(L.) merr. and l.m.perry	Flower	17:1	0.1	<25	n.d.
*Thymus vulgaris* L.	Herb	14:1	0.1	<25	n.d.
*Vaccinium myrtillus* L.	Fruit	2:1	1	79.9* ± 1.1	<25
*Vitis vinifera* L.	Seed (pomace)	15:1	1	<25	n.d.

**TABLE 3:** Results of glucose and fructose uptake studies with aqueous extracts, the used plant part, drug extract ratio, and tested concentration, which did not show any influence on cells in MTT assay. Only the plants that showed the strongest inhibition rates on glucose uptake were tested for fructose uptake inhibition. Regarding methanolic and aqueous extracts, the extract of a plant that showed the stronger inhibition on glucose absorption was repeated and tested for its impact on fructose uptake; mean value ± SD; *significant difference in *U*-test (*n* = 2–3; 12 replicates each; two-sided; *α* = 0.05); n.d. = not determined.

Scientific plant name	Used part of the plant	Drug extract ratio for aqueous extracts	Used aqueous extract concentration in mg/mL	Uptake inhibition of glucose in %	Uptake inhibition of fructose in %
*Adenophora triphylla* (thunb.) ADC.	Root	n.d.	1	<40	n.d.
*Allium sativum* L.	Bulb	2:1	1	<50	n.d.
*Aronia melanocarpa* michx. elliott	Fruit	3:1	1	<60	n.d.
*Artemisia dracunculus* L.	Leaf	5:1	1	<25	n.d.
*Brassica oleracea*L. “*Capitata alba*”	Leaf	2:1	0.1	<60	n.d.
*Camellia sinensis*(L.) kuntze (Assam)	Leaf	6:1	0.1	<25	n.d.
*Camellia sinensis*(L.) kuntze (Darjeeling)	Leaf	7:1	0.1	<25	n.d.
*Camellia sinensis*(L.) kuntze (gunpowder)	Leaf	5:1	0.1	<25	n.d.
*Camellia sinensis*(L.) kuntze (sencha)	Leaf	6:1	0.1	<25	n.d.
*Ceratonia siliqua* L.	Fruit	3:1	1	<25	n.d.
*Citrus limon* (L.) osbeck	Fruit skin	4:1	1	<40	n.d.
*Coffea arabica* L.	Green seed	5:1	1	<25	n.d.
*Cornus officinalis*siebold and zucc.	Fruit	n.d.	1	<65	n.d.
*Crataegus pinnatifida* bunge	Fruit	n.d.	1	<60	n.d.
*Cuminum cyminum* L.	Seed	5:1	0.1	<25	n.d.
*Cynara cardunculus* L.	Herb	3:1	0.1	<30	n.d.
*Eucommia ulmoides* oliv.	Bark	16:1	1	68.7* ± 13.2	<25
*Hibiscus sabdariffa* L.	Flower	2:1	0.1	<25	n.d.
*Ilex paraguariensis* a. st.-hil	Leaf	5:1	0.1	<25	n.d.
*Juglans regia* L.	Leaf	7:1	1	<25	n.d.
*Lycium chinense* mill.	Fruit	3:1	1	<75	n.d.
*Malus domestica*borkh. “*Gold en delicious*”	Fruit skin	3:1	1	57.5* ± 9.2	<25
*Melissa officinalis* L.	Leaf	4:1	0.01	<25	n.d.
*Mentha aquatica* L.	Leaf	6:1	1	<40	n.d.
*Momordica charantia* L.	Fruit	6:1	1	<25	n.d.
*Nigella sativa* L.	Seed	4:1	0.1	<25	n.d.
*Olea europaea* L.	Leaf	5:1	1	<25	n.d.
*Origanum creticum* L.	Leaf	4:1	0.1	<25	n.d.
*Panax ginseng* c.a. mey	Root	3:1	1	<25	n.d.
*Peumus boldus* molina	Leaf	n.d	1	<40	n.d.
*Potentilla aurea* L.	Herb	8:1	0.1	<25	n.d.
*Pueraria lobata* (willd.) ohwi	Root	4:1	1	<50	n.d.
*Punica granatum* L.	Fruit skin	3:1	0.1	<25	n.d.
*Rosa rugosa* thunb.	Flower	n.d	0.1	<30	n.d.
*Rosmarinus officinalis* L.	Leaf	5:1	0.1	<25	n.d.
*Salvia officinalis* L.	Leaf	5:1	0.1	<25	n.d.
*Sarcopoterium spinosum* (L.) spach	Root	28:1	1	<25	n.d.
*Syzygium aromaticum*(L.) merr. and l.m.perry	Flower	n.d.	0.1	<25	n.d.
*Thymus vulgaris* L.	Herb	6:1	1	<25	n.d.
*Vaccinium myrtillus* L.	Fruit	2:1	1	<80	n.d.
*Vitis vinifera* L.	Seed (pomace)	27:1	1	<40	n.d.

### General Handling of the Cell Line

Caco2 cells were purchased from Leibniz-Institut DSMZ-Deutsche Sammlung von Mikroorganismen und Zellkulturen GmbH (Braunschweig, Germany) and seeded in 24-well plates, which were acquired from Greiner Bio-One GmbH (Frickenhausen, Germany), for every experiment. As general medium DMEM (Dulbecco’s modified eagle medium) without phenol red containing 1% UltraGlutamine 1, both acquired from Lonza Cologne AG (Cologne, Germany) and 5 or 20% FBS derived from Bio&SELL GmbH (Nürnberg, Germany) for experiment or mother line, respectively, was used for the handling with Caco2 cells. Cells were washed once with PBS from Lonza Cologne AG (Cologne, Germany) for every medium change and washed twice before Trypsin (TrypLE™ Express without phenol red) from Gibco Life Technologies corporation (NY, United States) was applied to detach the cells for seeding. The cells were incubated at 37°C and 5% CO_2_-supply during the whole time. Passages 20–35 were used in the experiments. The buffers and solutions for cell culture were prepared with water obtained from the ultrapure water system LaboStar from Siemens AG Wasseraufbereitung (Barsbüttel, Germany).

### Glucose and Fructose Uptake in Caco2 Cells

For uptake studies, cells were seeded in a density of 400,000 cells per well. Confluence appeared 24 h after seeding and was controlled visually. The radiolabeled substrates 1 mCi/ml glucose (specific activity: 30 Ci/mmol) and 1 mCi/ml fructose (specific activity: 5 Ci/mmol) were obtained from Biotrend Chemikalien GmbH (Köln, Germany). Although fructose absorption into cells was determined by using 54 nM solution of radiolabeled substrate as final well concentration, the 2 nM radiolabeled glucose solution was spiked with 0.001 µM non-radiolabeled glucose, which was purchased from Merck KGaA (Darmstadt, Germany). For uptake studies with extracts, cells were used at day three and day fifteen after confluence for glucose and fructose, respectively. On the day of the experiment, cells were washed once with 2 ml buffer containing 20 mM Hepes [4-(2-hydroxyethyl)-1-piperazineethanesulfonic acid], which was obtained from Carl Roth GmbH + Co. KG (Karlsruhe, Germany) and 150 mM NaCl purchased from Fisher Scientific GmbH (Schwerte, Germany). The pH of the buffer was adjusted with sodium hydroxide from Merck KGaA (Darmstadt, Germany) using a 766 Laboratory pH meter from Knick Elektronische Messgeraete GmbH & Co. KG (Berlin, Germany) to 7.4. After 1 h starving incubation with Hepes-NaCl buffer at 37°C, substrate or substrate-extract solution were added to the cells and incubated at 37°C. After 1 h, the buffer was discarded, the cells were washed once with 1 ml ice-cold Hepes-NaCl buffer, and 500 µL Hepes-NaCl solution containing 1% Triton X 100 was added to each well. The samples were taken from the well plates, added to a sufficient volume of scintillation cocktail in scintillation tubes, both purchased from Hidex (Mainz, Germany), mixed for 20 s using a vortexer from Heidolph Instruments GmbH & Co. KG (Schwabach, Germany), and measured with a β-Counter from Hidex (Mainz, Germany). The plant extracts, which showed the strongest inhibition on intestinal glucose transporters, were repeated and tested for their influence on fructose uptake with a focus on methanolic extracts. Randomly, well plates, which were cultivated and treated under the same conditions as cells used for uptake studies, were tested in Hoechst Assay as described in the Supplementary Material to determine variations depending on the procedure.

### Statistical Evaluation and Use of Software

All statistical tests were performed using Microsoft Excel. Normal distribution was established *via* Shapiro–Wilk test manually before using Excel’s tool “Data analysis” for provision of the *F*-test and the *t*-tests. If the data was not normally distributed, Mann–Whitney *U* test was applied for further statistical analysis. IC_50_ data was calculated with GraphPad Prism 5.0.

## Results

### Uptake Studies with Glucose and Fructose

**FIGURE 1:**
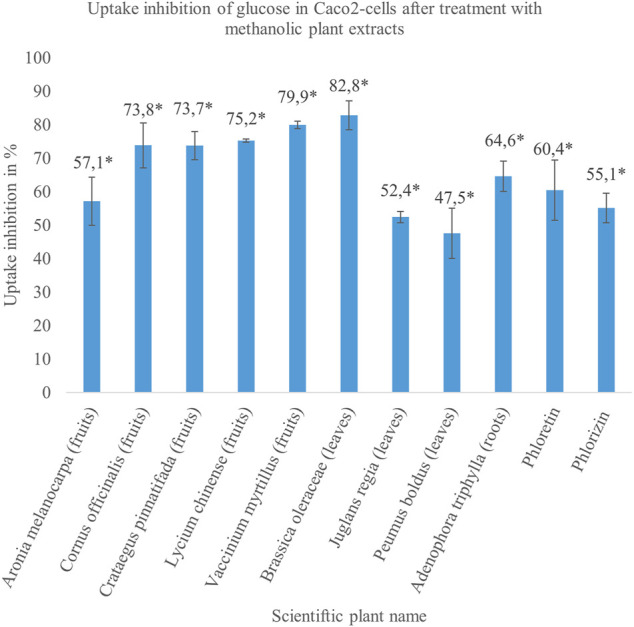
The results of the methanolic plant extracts, which reduced glucose uptake between 40 and 80% in treated cells significantly compared to untreated cells in a concentration of 1 mg/ml. The extracts were made from different parts of the plants enclosed in brackets. The solutions of 100 µM phloretin and 100 µM phlorizin were used as positive controls and inhibited glucose uptake in Caco2 cells by approximately 60%. The illustrated results refer to the data in Table 2. Mean value ± SD. * Significant difference in *U*-test (*n* = 2 with 12 replicates each; two-sided, *α* = 0.05).

In order to prevent decreased metabolic activity due to the use of toxic extracts, methanolic and aqueous plant preparations were tested in MTT assay and the concentration, which did not reduce cell viability significantly, was used for uptake studies as shown in Tables 2, 3. According to the results obtained from Hoechst Assay that showed an average coefficient of variation of 21.7% within 14 randomly tested well plates, uptake inhibition of plant extracts was distinguishable from general fluctuations in the procedure for values >25%. In every experiment, a control without extract was running and served as 100% uptake control. After correction of values by subtracting the blank, uptake inhibition was calculated as the difference between control and sample. The obtained values of monosaccharide uptake inhibition in Caco2 cells treated with methanolic and aqueous extracts are reported in Tables 2, 3, respectively. Only the herbal drugs that showed the strongest inhibition rates on glucose uptake were tested for fructose uptake inhibition. For some of the plants, methanolic and aqueous extracts decreased glucose uptake significantly. However, only the extract of a plant that showed stronger inhibition on glucose absorption was repeated and tested for its impact on fructose uptake as described for the following plants. Additionally, the focus for further studies was on methanolic extracts as they naturally contain fewer monosaccharides, which could interfere with the measurement. The methanolic extracts made from the fruits of *Aronia melanocarpa* (uptake inhibition: 57.1%), *Cornus officinalis* (uptake inhibition: 73.8%), *Crataegus pinnatifida* (uptake inhibition: 73.7%), *Lycium chinense* (uptake inhibition: 75.2%), and *Vaccinium myrtillus* (uptake inhibition: 79.9%); the leaves of *Brassica oleracea* (uptake inhibition: 82.8%), *Juglans regia* (uptake inhibition: 52.4%), and *Peumus boldus* (uptake inhibition: 47.5%); and the roots of *Adenophora triphylla* (uptake inhibition: 64.6%) indicated a strong and reproducible inhibitory potential between 40 and 80% on intestinal glucose transporters as visualized in Figure 1. Seven of these extracts showed higher uptake inhibition rates than the two controls phloretin (uptake inhibition: 60.4%) and phlorizin (uptake inhibition: 55.1%). A queous extracts made from the bark of *Eucommia ulmoides* (uptake inhibition: 68.7%) and the fruits of *Malus domestica* (uptake inhibition: 57.5%) inhibited glucose uptake significantly and reproducibly. Additionally, for the methanolic extract of *Brassica oleracea* and the aqueous extract of *Eucommia ulmoides*, IC_50_ was calculated as 0.0904 and 0.4285 mg/ml, respectively. These extracts decreased glucose uptake in a dose-dependent manner. The methanolic extracts of *Juglans regia* (uptake inhibition: 30.2%) and *Peumus boldus* (uptake inhibition: 32.6%) inhibited the fructose uptake significantly.

## Discussion

According to the results of the present study, the observed hypoglycemic effects in animal and/or human studies after treatment with preparations of *Adenophora triphylla* , *Aronia melanocarpa*, *Brassica oleracea*, *Cornus officinalis* , *Crataegus pinnatifida*, *Eucommia ulmoides*, *Juglans regia* , *Lycium chinense*, *Malus domestica*, *Peumus boldus*, and *Vaccinium myrtillus* as listed in Table 1 possibly relate to the inhibition of intestinal transporters. The methanolic plant extracts made from the leaves of *Juglans regia* and *Peumus boldus* decreased glucose as well as fructose uptake possibly related to inhibition of GLUT2, which is able to transport both monosaccharides. These findings show that the traditional use of *Brassica oleracea*, *Cornus officinalis*, *Eucommia ulmoides*, *Juglans regia*, *Lycium chinense*, *Malus domestica*, and *Vaccinium myrtillus* and their preparations as antidiabetic remedy and adjuncts to conventional treatments in the therapy of Diabetes mellitus is reasonable. Extracts of these plants show inhibitory activity on intestinal glucose and fructose transporters resulting in lower blood concentrations of the monosaccharides and reduced concomitant health problems.

In literature, there is not much comparable data available, because the inhibition of intestinal transporters by crude plant extracts in Caco2 cells has not been investigated very well (Schreck and Melzig, 2018). Kim et al. determined a moderate inhibition of glucose uptake in Caco2 cells for methanolic extracts of *Adenophora triphylla* (uptake inhibition: 13.4%) and *Cornus officinalis* (uptake inhibition: 16.4%), but other results as for *Crataegus pinnatifida* (no uptake inhibition) are not consistent with our findings (Kim et al., 2011). Zhang et al. confirmed an uptake inhibition of 26.3% for *Eucommia ulmoides* (Zhang et al., 2015). Manzano et al. only tested specific polyphenol-rich fractions of *Malus domestica* (Manzano and Williamson, 2010), which showed inhibitory activity on intestinal glucose transporters, but it is not directly comparable with our work. Discrepancies between results from different studies probably depend on different preparations of the extracts, the use of modified substrates, and/or an insufficient validation of the cell model. We determined the expression of relevant transporters over three weeks in Caco2 cells and adapted our experimental settings in accordance with the ensured presence of functional transporters (Schreck and Melzig, 2019). Buchholz et al. examined whether extracts of the traditionally used plants inhibit cleavage of polysaccharides and lipids by affecting lipase and α-amylase activity (Prasain et al., 2012). The methanolic extracts of *Aronia melanocarpa*, *Peumus boldus*, and *Vaccinium myrtillus* showed strong lipase and α-amylase inhibition as well as inhibition of intestinal monosaccharide transporters in our studies. This multi-target effect of the mentioned plant preparations describes a partial, but multiple drug action and is proposed to exceed drugs with single-target effects. Based on synergistic mechanisms, the plants that affect various targets to alleviate monosaccharide uptake show therapeutic advantages concerning higher intended and lower adverse effects (Csermely et al., 2005). Since the plants, especially the fruits, contain sugars themselves, it cannot be totally excluded that a part of the inhibition is a “pseudo-inhibition” and comes from the displacement of the radioactive substrates by non-radioactive monosaccharides during absorption processes. For this reason, the focus of this work was on methanolic extracts that naturally contain fewer monosaccharides. Commonly, the discussed constituents of the plants with inhibitory effects on intestinal transporters are polyphenolic compounds as shown in Table 1. Particularly, the chalcones phlorizin and phloretin (Debnam and Levin, 1975; Zheng et al., 2012; Raja and Kinne, 2015), which are often used as positive controls in transport studies, the flavonoids quercetin, fisetin, and myricetin (Kwon et al., 2007) and the tannins (-)-epigallocatechin gallate, (-)-epigallocatechin, and (-)-epicatechin gallate seem to act as inhibitors of intestinal, monosaccharide transporters (Johnston et al., 2005). Further studies are planned to investigate which fraction of the extracts, or more specifically, which group of compounds are responsible for the inhibition of the intestinal glucose and fructose transporters in this study.

## Data Availability

The original contributions presented in the study are included in the article/Supplementary Material, and further inquiries can be directed to the corresponding author.
