# Supplementary material for: Traditionally Used Plants in the Treatment of Diabetes Mellitus: Screening for Uptake Inhibition of Glucose and Fructose in the Caco2-Cell Model
Source: Front Pharmacol. 2021 Aug 20;12:692566. doi: 10.3389/fphar.2021.692566 (PMC8417609; doi:10.3389/fphar.2021.692566)
Supplement: Supplementary file 1 [file DataSheet1.PDF]

Supplementary Material

1 Supplementary Data

1.1 MTT-assay

Cells were seeded in a density of 30,000 cells each well and incubated for 48 h. After that, methanolic and aqueous extract solutions prepared as described previously (1.00, 0.10 and 0.01 mg/mL) were added to the wells before incubation for another 24 h. Subsequently, the MTT, which was obtained from Carl Roth GmbH+Co. KG (Karlsruhe, Germany), was solved in DPBS (5 mg/mL), added to the wells and the well plates were incubated for 2 h at 37°C in a 5 % CO_2-atmosphere. The medium was discarded and replaced by a sufficient volume of DMSO. After shaking for 5 min, the absorbance was measured by a Tecan Infinite F200 Microplate reader purchased from Tecan Group Ltd. (Männedorf, Switzerland) at 560 nm and 620 nm. Cells treated with DPBS instead of extracts were considered as 100 % viability control and cells treated with 1 % Triton X 100, which was purchased from SIGMA-ALDRICH Chemie GmbH (Steinheim, Germany), in DPBS applied as negative controls. Additionally, all extracts were also running without cells as a reference to determine the absorbance from every extract itself.

1.2 Hoechst-Assay

The fluorescent dye Hoechst 33258 was used to validate the system by determining the differences within well plates regarding the amount of cells. The assay was performed with the FluoReporter™ Blue Fluorometric dsDNAQuantitation kit from Life Technologies Corporation (Eugene, USA). One supplementary well plate was seeded with the same cell suspension as the well plates for the uptake studies and treated the same way until day of experiment. Instead of using the cells for uptake studies, the medium of these cells was replaced with ultrapure water and the cells were incubated for 1 h at 37°C in a 5 % CO_2-atmosphere, before they were stored in a refrigerator at -20°C overnight. After 3 freezing-thaw cycles, cells were lysed in cOmplete Lysis-M from Roche Diagnostics GmbH (Mannheim, Germany) for at least 5 min, homogenized and an appropriate volume was transferred to a black 96F-Nunclon™-well plate from Fisher Scientific GmbH (Schwerte, Germany). The reagent was added to each well with a multichannel pipette according to the protocol of the kit and after excitation at 360 nm, the fluorescence was measured at 460 nm by a Tecan Infinite F200 Microplate reader.
